# Protective and Healing Effects of Zinc L-Carnosine on the Oral Mucosa: A Systematic Review and Meta-Analysis

**DOI:** 10.3390/dj14070408

**Published:** 2026-07-05

**Authors:** Pierpaolo De Francesco, Paolo Vescovi, Giuseppe Pedrazzi, Ilaria Giovannacci

**Affiliations:** 1 Oral Medicine and Oral Laser Surgery Unit, Department of Medicine and Surgery, University of Parma, Via Gramsci 14, 43125 Parma, Italy; pierpaolo.defrancesco@unipr.it (P.D.F.); paolo.vescovi@unipr.it (P.V.); 2 Unit of Neuroscience, Department of Medicine and Surgery, Plesso Biotecnologico Integrato, University of Parma, 43125 Parma, Italy; giuseppe.pedrazzi@unipr.it; 3 Interdepartmental Center of Robust Statistics (Ro.S.A.), University of Parma, 43125 Parma, Italy; 4 UniCamillus-Saint Camillus International University of Health Sciences, 00184 Rome, Italy

**Keywords:** oral mucosa, wound healing, oral mucositis, oral surgery, zinc L-carnosine

## Abstract

**Background/Objectives**: Oral mucosal injury is a frequent complication in oncologic and surgical settings, significantly affecting patient quality of life. Zinc L-carnosine (ZnC) is a cytoprotective compound with anti-inflammatory, antioxidant, and epithelial reparative properties. This systematic review evaluated its protective and healing effects on oral mucosa. **Methods**: A systematic search followed PRISMA guidelines was conducted across PubMed, Scopus, Web of Science, and Cochrane Library (2015–2026). Randomized and non-randomized controlled studies assessing ZnC in patients with or at risk of oral mucosal injury were included. Risk of bias was evaluated using RoB 2 and ROBINS-I tools. Meta-analyses were conducted under both common- and random-effects models. The certainty of evidence was evaluated according to the GRADE guidelines. **Results**: Eight studies (*n* = 544) were included. Six non-randomized studies showed moderate or serious risk of bias, while randomized trials presented some concerns. ZnC was administered in different formulations, including mouthwashes, lozenges, and mucoadhesive suspensions based on sodium alginate, polyacrylic acid, and carboxyvinyl polymer, and across different clinical settings. Meta-analysis showed a reduced incidence of severe oral mucositis (grade ≥ 3) under the Common Effect model (OR 0.48; 95% CI 0.32–0.72), although statistical significance was not maintained under random-effects models (OR 0.44; 95% CI 0.18–1.06). Similar results were observed for grade ≥ 2 mucositis. According to the GRADE assessment, the certainty of evidence was low for oral mucositis outcomes and very low for oral mucosal healing. Only one study suggested improved surgical wound healing. No serious adverse events were reported. **Conclusions**: ZnC may support oral mucosal protection and healing, particularly in preventing oral mucositis. However, substantial heterogeneity and limited high-quality randomized evidence restrict the strength of conclusions. Further well-designed randomized trials are needed.

## 1. Introduction

The oral mucosa represents a critical interface between the external environment and the host, providing first-line protection against potential pathogens, exogenous substances, and airborne allergens through its physical and microbiological immune barrier functions [1,2]. Damage to the oral mucosa is a common and clinically relevant condition in various medical and dental contexts, including cancer therapies, oral and maxillofacial surgery, and inflammatory or traumatic conditions [3]. In particular, Oral Mucositis (OM) induced by chemotherapy and radiotherapy and oral surgical wounds are distinct clinical manifestations characterized by the alteration of the integrity of the mucosal barrier, and are associated with discomfort and pain for the patient [4]. OM is not only a local problem but can also have very debilitating repercussions for patients, often characterized by intense pain, difficulty eating properly, malnutrition, and, in the most severe cases, the need for parenteral nutrition [5]. In addition, this condition is often associated with increased hospitalization and higher healthcare costs due to its difficult and complex management [6]. It is a very common complication that occurs in about 40% of patients undergoing chemotherapy, rising to 60–85% in patients who are candidates for hematopoietic stem cell transplantation (HSCT), reaching even higher percentages (90%) in patients with head and neck cancer undergoing radiotherapy (RT) or radiochemotherapy (RCT). Approximately 11–19% of patients with OM are forced to delay or suspend their cancer treatment, with possible repercussions on their prognosis [7,8]. Unfortunately, the management of OM remains a very complex and a clinically significant challenge. A universally accepted gold standard is lacking, and treatment is largely based on oral care support measures [9]. Restoration of the oral mucosa following injury represents a significant clinical challenge due to the complex anatomy of the oral cavity and the essential protective and functional roles of the mucosal barrier [10]. This process progresses through overlapping phases: hemostasis, inflammation, proliferation, and remodeling, that are essential for successful regeneration [11]. Dysregulation of the inflammatory response negatively affects healing outcomes, favoring the development of postoperative complications, and to minimize these adverse events, current evidence supports a variety of therapeutic strategies aimed at enhancing oral wound repair. In this context, increasing interest has been directed toward agents capable of supporting oral mucosal integrity and repair by modulating inflammatory responses, oxidative stress, and epithelial regeneration. Many of these strategies rely on the local application of agents or devices capable of adapting to the humid oral environment and modulating the wound microenvironment to promote tissue regeneration [12]. In clinical practice, topical formulations such as gels, pastes, and ointments are widely used to deliver bioactive compounds directly to the oral mucosa, while adjunctive physical modalities, including low-level laser therapy, have been explored to further enhance healing responses [13,14]. Zinc L-carnosine (ZnC) is a chelated compound composed of zinc and L-carnosine that has been investigated for its potential role in mucosal protection and tissue repair [15]. The biological rationale for its use is based on the complementary properties of its components, which have been associated with epithelial barrier preservation, modulation of inflammatory responses, and antioxidant activity [16].

Zinc is an essential element involved as a cofactor of many enzymatic pathways such as cell proliferation, immune function, and wound healing, while L-carnosine is a naturally occurring dipeptide (β-alanyl-L-histidine) with cytoprotective and antioxidant properties [17]. Experimental evidence suggests that ZnC may support epithelial repair processes by limiting oxidative stress and by contributing to cellular mechanisms involved in re-epithelialization [18].

Through these barrier-protective, reparative, and antioxidant mechanisms, ZnC represents a biologically plausible candidate for supporting oral mucosal protection and healing in conditions characterized by epithelial damage [15,16]. Despite the growing number of clinical studies investigating the use of ZnC in conditions involving mucosal injury, the available evidence remains fragmented across heterogeneous clinical settings, study designs, and outcome measures. In particular, no systematic review has specifically focused in vivo human evidence regarding the protective and healing effects of zinc L-carnosine on the oral mucosa.

Therefore, the aim of the present systematic review is to evaluate the available clinical evidence on the role of zinc L-carnosine in oral mucosal protection and healing with particular attention to the preventive and therapeutic effect on radio- and chemotherapy- induced oral mucositis.

## 2. Materials and Methods

### 2.1. Protocol Development and Eligibility Criteria

This systematic review was organized and reported according to the recommendations of the PRISMA (reporting items for systematic reviews and meta-analysis) 2020 Statement [19]. The PRISMA 2020 checklist is provided as Supplementary Material.

The review protocol was accepted and registered with PROSPERO, the International Prospective Register of Systematic Reviews, maintained by the National Institute for Health Research at the University of York’s Centre for Reviews and Dissemination (CRD420261299697).

All in vivo studies that utilized zinc L-carnosine (ZnC) to investigate its role in mucosal protection and repair were included.

The following focused question was phrased: What is the clinical evidence supporting the use of zinc L-carnosine for the protection and healing of the oral mucosa?

Inclusion criteria for articles were based on the following PICOS:

P (Problem/Population): All in vivo studies that evaluate patients with or at risk of oral mucosal damage.

I (Intervention): Administration of ZnC (polaprezinc), in any formulation or dose.

C (Comparison): Use of ZnC was compared with any type of treatment or no treatment.

O (Outcome): Oral mucosal protection and healing.

S (Study design): Any type of study design.

### 2.2. Information Sources and Search

Relevant studies published between January 2015 and February 2026 were identified through searches of the PubMed, Scopus, Web of Science, and Cochrane Library electronic databases. Given the limited and highly specific literature available on zinc L-carnosine in oral mucosal conditions, a broad keyword-based search strategy was adopted to maximize sensitivity. The search strategy utilized the following terms:

(“Zinc L-Carnosine” OR “Zinc-L-Carnosine” OR “Zinc Carnosine” OR “Polaprezinc”)

AND

(“mucositis” OR “stomatitis” OR “oral wound*” OR “oral surgical wound* “OR “oral surgery” OR “wound healing”)

During the database searches no automatic filters were applied for humans or language. Instead, restrictions to human studies and English language were applied during the manual screening stage, as specified in the inclusion criteria. In addition, the reference lists of all included studies were manually screened to identify potentially relevant articles.

### 2.3. Study Selection and Data Collection

To determine the inclusion of articles in the review, a double evaluation was carried out: first, the titles and abstracts were examined by two independent reviewers (PDF and IG); then, articles deemed potentially relevant were subjected to a thorough full-text analysis. Data extraction was also performed independently by the same two reviewers (PDF and IG).

In order to minimize the risk of excluding relevant evidence, abstracts with poorly defined results were included in the full-text review.

Inclusion criteria for the title and abstract analysis were the following:

- Manuscripts published in English between January 2015 and January 2026 in order to focus on contemporary clinical evidence and modern formulations of zinc L-carnosine
- Articles with available full text
- In vivo human studies evaluating the protective and/or healing effects of zinc L-carnosine (polaprezinc)

Exclusion criteria for title and abstract analysis were:

- Studies involving non-oral mucosal sites only (e.g., gastrointestinal, nasal, or genital mucosa)
- Studies evaluating zinc compounds other than zinc L-carnosine
- Not original study (abstract, guidelines, and letters)
- Case reports or case series with fewer than 10 cases
- In vitro studies
- Animal studies

Full-text articles of all potentially relevant studies were retrieved and independently assessed by two reviewers (PDF and IG) against the inclusion criteria. Discrepancies were resolved through discussion with a senior reviewer (PV). The same exclusion criteria were applied to the full-text analysis, together with absence of reporting of any of the studied outcomes.

Primary outcomes included:

1. Oral mucosal protection:
  - Incidence of clinically relevant Oral Mucositis (WHO/CTCAE grade ≥ 2)
  - Incidence of severe OM (WHO/CTCAE grade ≥ 3)
2. Oral mucosal healing:
  - Resolution of mucosal injury: Time to clinical resolution
  - Tissue repair quality (in surgical settings): validated wound healing scores

Secondary outcomes included: pain; oral function; adverse events; discomfort.

### 2.4. Risk of Bias Assessment

To assess the methodological quality and risk of bias of the included studies, randomized controlled trials [20,21] were evaluated using the Cochrane Risk of Bias tool (RoB 2), while non-randomized studies [6,17,22,23,24,25] were assessed using the ROBINS-I (Risk of Bias in Non-randomized Studies of Interventions).

### 2.5. Data Synthesis and Statistical Analysis

Data synthesis was performed in accordance with PRISMA recommendations for quantitative evidence synthesis. Meta-analyses were conducted separately for two clinically relevant endpoints: incidence of grade ≥ 2 oral mucositis and incidence of grade ≥ 3 oral mucositis.

For each included study, 2 × 2 contingency tables were reconstructed using the number of events and total participants in the intervention and control groups. The effect measure was the odds ratio (OR) with corresponding 95% confidence intervals (95% CIs). Odds ratios were log-transformed prior to pooling, and standard errors were derived from the inverse of the within-study variance.

Pooled effect estimates were calculated using the inverse-variance method under both fixed-effect and random-effects models. The fixed-effect model assumed a common true effect across studies, while the random-effects model accounted for potential between-study variability arising from clinical or methodological heterogeneity.

Statistical heterogeneity was assessed using Cochran’s Q test and quantified using the I^2^ statistic, with values of approximately 25%, 50%, and 75% interpreted as low, moderate, and high heterogeneity, respectively. For random-effects analyses, the between-study variance (τ^2^) was primarily estimated using the Paule–Mandel (PM) estimator. As a sensitivity analysis, restricted maximum likelihood (REML) estimation was additionally performed to evaluate the robustness of pooled estimates to different heterogeneity estimators.

Given the limited number of included studies, the Hartung–Knapp adjustment was applied to random-effects models to provide more conservative confidence intervals and reduce the risk of type I error inflation.

Forest plots were generated to visually display individual study estimates and pooled effect sizes under both fixed-effect and random-effects models, including corresponding 95% confidence intervals and study weights.

All statistical analyses were performed using the meta package in R (R version 4.3.1; R Foundation for Statistical Computing, Vienna, Austria). All tests were two-sided, and statistical significance was defined as *p* < 0.05. Publication bias was explored using funnel plots and Egger’s regression test for both meta-analytic outcomes. Given the limited number of studies included in each analysis, these assessments were considered exploratory and interpreted with caution. The certainty of evidence was evaluated according to the GRADE (Grading of Recommendations Assessment, Development and Evaluation) assessment.

## 3. Results

### 3.1. Study Selection

A total of 246 citations of articles published in English between January 2015 and February 2026 were identified for inclusion in the review. After removing duplicates, 162 articles remained for analysis. A further 154 articles were excluded following the screening of titles and abstracts. Full-text analysis was conducted on the remaining eight articles, and all were included in the qualitative synthesis [6,17,20,21,22,23,24,25]. 

### 3.2. PRISMA Flow Diagram

**Figure 1:**
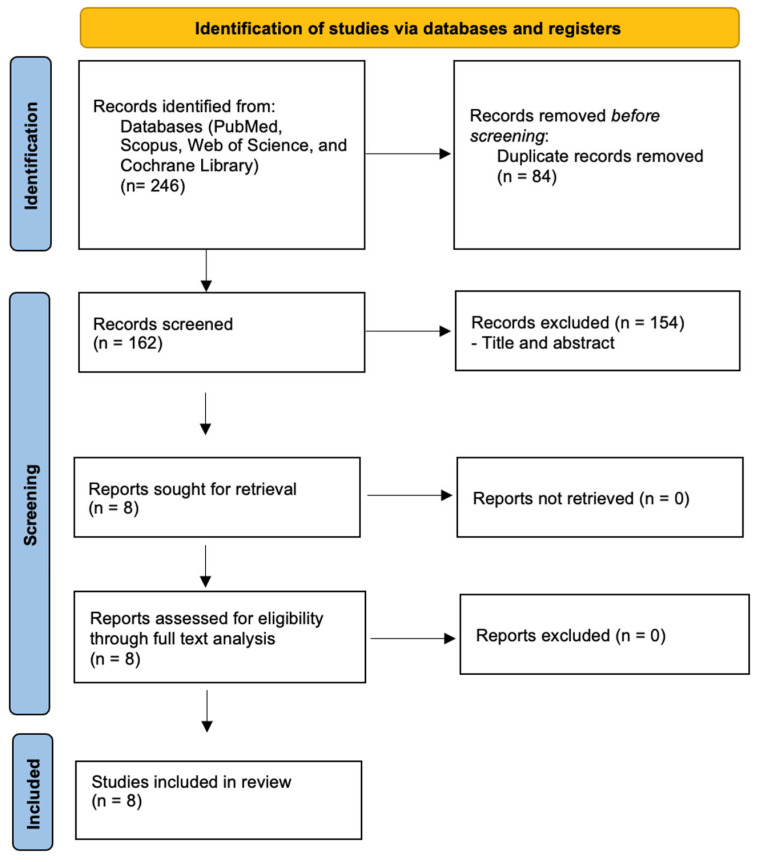
PRISMA Flow chart.

The study selection process is detailed in the PRISMA 2020 flow diagram, now presented as Figure 1.

### 3.3. Results of Risk of Bias Assessment

**Figure 2:**
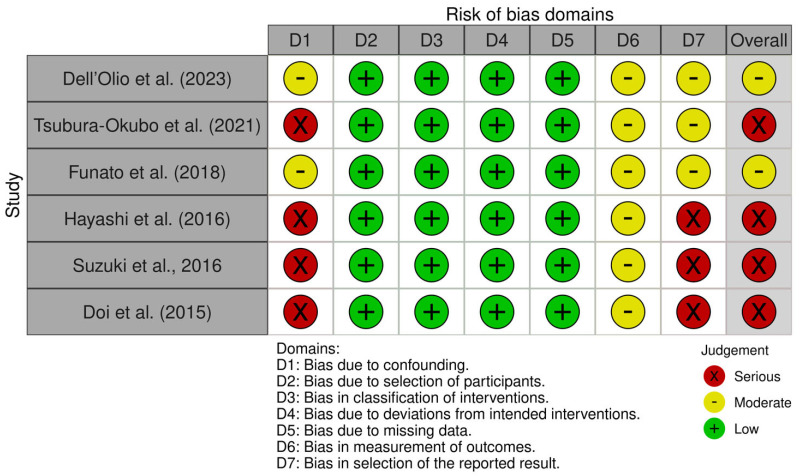
ROBINS-I (Risk of Bias in Non-randomized Studies of Interventions) [6,17,22,23,24,25].

The risk-of-bias assessment for the non-randomized studies (ROBINS-I) is summarized in Figure 2. Among the six studies, four were classified to be at serious risk of bias and two at moderate risk of bias.

**Figure 3:**
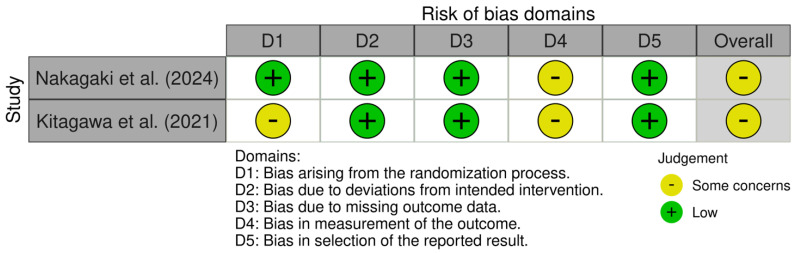
Rob 2 (Cochrane Risk of Bias tool) [20,21].

The risk-of-bias assessment for the randomized controlled trials (RoB 2) is presented in Figure 3. Both studies were judged to have overall risk of bias defined as “some concerns.” Several studies used retrospective designs and historical control groups, increasing the potential for confounding. In addition, outcomes such as oral mucositis severity and mucosal healing were often assessed clinically without blinding of the evaluators, which may have influenced outcome measurement.

### 3.4. Level of Evidence

The analysis included eight in vivo studies on humans, all of which were assessable according to the Oxford Centre for Evidence-Based Medicine (OCEBM) 2011 Levels of Evidence [6,17,20,21,22,23,24,25]. 

The level of evidence was 2 for two studies [17,20] and 3 for six studies [6,21,22,23,24,25].

### 3.5. Population and Studies Characteristics

A total of eight in vivo human studies were included in the present study. Two were randomized controlled trials, while six were non-randomized prospective or retrospective studies. The studies investigated the use of zinc L-carnosine in heterogeneous clinical settings, including hematopoietic stem cell transplantation, high-dose chemotherapy (CT), radiotherapy or chemoradiotherapy (RT/CRT) for head and neck cancer (HNC), and oral surgical wounds.

The study population consisted of 544 patients. Patient age ranged from 1 to 90 years, reflecting the inclusion of both pediatric and adult populations (Table 1). In all selected studies [6,17,20,21,22,23,24,25], patients were generally excluded if they presented systemic medical conditions known to impair oral mucosal healing, such as uncontrolled systemic diseases, severe comorbidities, or pre-existing oral mucosal lesions prior to treatment. Zinc L-carnosine was administered using different formulations, including mouthwashes [20], lozenges [17,23], and sodium alginate-based/polyacrylic acid/carboxyvinyl polymer-based suspensions [6,21,22,24,25], designed to enhance mucosal adhesion, either as a preventive intervention or as therapeutic treatment. Control groups received standard oral care (routine oral management or no specific prophylactic treatment) or control mouthwashes (e.g., sodium bicarbonate or azulene gargle).

### 3.6. Oral Mucosal Protection: Incidence and Severity of Oral Mucositis

The protective effect of using ZnC in various formulations was assessed by evaluating clinical outcomes, such as the incidence of oral mucositis (OM), as defined in each study, and the incidence of severe OM (Grade ≥ 3). The assessment of mucositis grade was performed using the Common Terminology Criteria for Adverse Events (CTCAE) scale in five studies [6,17,21,23,25], and the World Health Organization (WHO) scale in two studies [20,24] (Table 2).

Studies [6,24] evaluating patients with HNC undergoing RT or CRT have shown that prophylactic administration of zinc L-carnosine is associated with a lower incidence of severe OM (Grade ≥ 3) compared to standard oral care, such as azulene gargle, indicating a protective effect against severe mucosal lesions during RT/CRT.

In the context of HSCT, ZnC has demonstrated a protective effect in most studies, although results varied depending on study design and formulation. Kitagawa et al. [21] showed that prophylactic administration of ZnC compared to delayed administration significantly reduced the incidence of grade ≥ 2 OM (22.0% vs. 44.7%, *p* = 0.025), while no significant difference was observed for grade ≥ 3 (14.6% vs. 10.6%). Tsubura-Okubo et al. [23] reported a significantly lower incidence of severe OM (grade ≥ 3) in patients treated with an adhesive ZnC formulation compared to standard oral therapy (15.2% vs. 36.6%, *p* = 0.008). Hayashi et al. [17] showed a statistically significant reduction (*p* < 0.01) in the incidence of OM (grade ≥ 2), which occurred in 22.6% of patients treated preventively with zinc L-carnosine suspension and in 12.5% of those treated with lozenges, compared to 73.7% in the control group. In the same study, the incidence of severe OM (grade ≥ 3) was lower in the groups treated with ZnC (3.2–6.3%) than in the control group (21.1%). In the pediatric population undergoing HSCT, Funato et al. [24] reported a reduction in incidence of severe OM (grade ≥ 3), which occurred in 20% of subjects treated with ZnC compared to 83.3% in the azulene gargle group (*p* = 0.035). Only one RCT, conducted by Nakagaki et al. [20], which used a ZnC mouthwash, showed no significant difference in the incidence of severe OM (grade ≥ 3), compared to the sodium bicarbonate mouthwash group.

**Figure 4:**
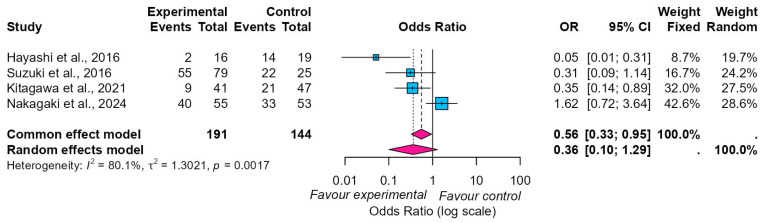
Forest plot of severe (grade ≥ 3) oral mucositis. Seven studies (*n* = 532). Common-effect model: OR 0.48 (95% CI 0.32–0.72); random effects model: OR 0.44 (95% CI 0.18–1.06). Heterogeneity: I^2^ = 56.1% [6,17,20,21].

Seven studies were included in the meta-analysis regarding the incidence of severe (≥3) mucositis, for a total of 532 observations. The odds ratio (OR) calculated with the Common Effects Model resulted statistically significant (*p* = 0.0004) in favour of the test group. Under the common-effect model, ZnC administration was associated with a lower incidence of severe OM (Figure 4). However, this finding was not confirmed under the random-effects model and should therefore be interpreted with caution.

**Figure 5:**
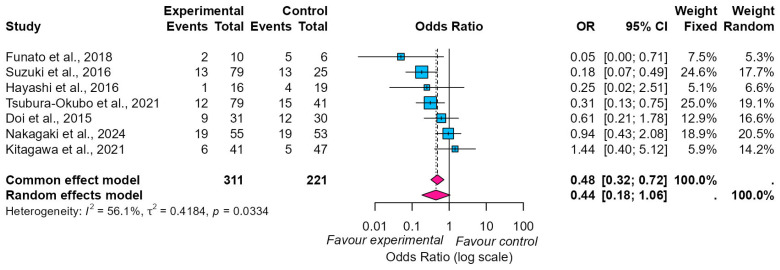
Forest plot of moderate (grade ≥ 2) oral mucositis. Four studies (*n* = 335). Common-effect model: OR 0.56 (95% CI 0.33–0.95); random effects model: OR 0.36 (95% CI 0.10–1.29). Heterogeneity: I^2^ = 80.1% [6,17,20,21,23,24,25].

Regarding the analysis of the incidence of moderate (≥2) OM, four studies were included in the meta-analysis for a total of 335 observations. In this case too, the OR calculated with the Common Effect model revealed a statistically significant difference in favour of the group in which ZnC was administered for preventive purposes (*p* = 0.0302) (Figure 5).

For both analyses, statistical significance was not maintained under the random-effects model. This finding likely reflects the substantial clinical and methodological heterogeneity among the included studies, including differences in patient populations, clinical settings, study designs, ZnC formulations, treatment strategies, and outcome assessment methods.

### 3.7. Publication Bias Assessment

**Figure 6:**
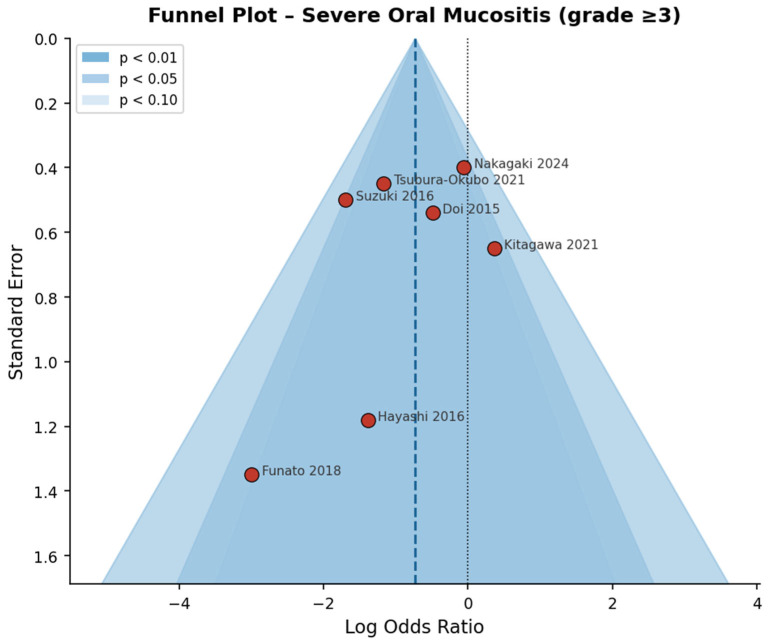
Funnel plot for severe oral mucositis (grade ≥ 3) [6,17,20,21,23,24,25].

**Figure 7:**
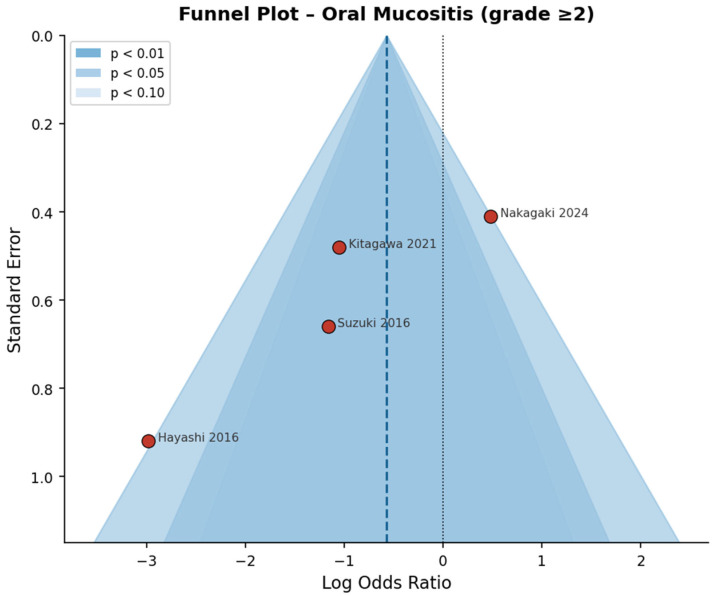
Funnel plot for moderate oral mucositis (grade ≥ 2) [6,17,20,21].

Publication bias was explored using funnel plots and Egger’s regression test for both meta-analytic outcomes (Figure 6 and Figure 7). For severe oral mucositis (grade ≥ 3), Egger’s regression test did not detect statistically significant asymmetry (*p* = 0.412). Similarly, for oral mucositis grade ≥ 2, no statistically significant asymmetry was detected (*p* = 0.125). Visual inspection of the funnel plots did not reveal marked asymmetry for either outcome.

However, because only seven and four studies were available for the grade ≥ 3 and grade ≥ 2 analyses, respectively, both funnel plot inspection and Egger’s regression test should be considered exploratory. These methods are recognized to have limited reliability and low statistical power when fewer than ten studies are included; therefore, publication bias cannot be reliably excluded.

### 3.8. Oral Mucosal Healing

Evidence regarding oral mucosal healing was limited and derived from a small number of studies, which assessed healing using heterogeneous outcomes (Table 3). Direct evidence was available from only one study [22], which evaluated post-extraction wound healing in healthy individuals, while the remaining studies reported indirect indicators of mucosal recovery.

The study of Dell’Olio et al. [22] showed direct evidence of oral mucosal healing in oral surgical wounds following wisdom tooth extraction, where zinc L-carnosine mouthwash was associated with significantly higher healing index (MHI) at 21 days (5.2 ± 1.3 vs. 4.7 ± 1.8, *p* = 0.026) and at 28 days (5.9 ± 0.3 vs. 5.4 ± 1.1, *p* = 0.048), while no significant differences were observed during the initial healing phase (7 and 14 days). Furthermore, the percentage healing index (PHI) was significantly higher in the ZnC group at 21 days (85.5% vs. 80.4%; *p* = 0.029) and 28 days (95.1% vs. 87.9%; *p* = 0.007).

In addition, indirect indicators of mucosal recovery were evaluated in patients treated with RT or CRT for HNC and in patients undergoing HSCT. In the study by Doi et al. [25], the administration of a ZnC mouthwash was associated with a shorter time to clinical resolution of radiation-induced oral mucositis compared to the control group. In the study of Kitagawa et al. [21], in which ZnC- based lozenge was started after the onset of grade 2 mucositis, the incidence of severe mucositis (OM ≥ 3) was 10.6%. In the context of patients undergoing HSCT, the study by Tsubura-Okubo et al. [23] reported significantly lower rates of oral pain and dysgeusia in the PPAA group compared with standard oral care during the acute treatment phase (oral pain: 26.6% vs. 75.6%; dysgeusia: 13.9% vs. 36.6%), suggesting a less severe clinical course of oral mucosal involvement.

### 3.9. Secondary Outcomes: Pain, Oral Function, and Adverse Events

Several studies reported secondary outcomes related to pain, oral function, and adverse events, although assessment methods and reporting were heterogeneous.

In studies evaluating pain-related symptoms, the administration of ZnC was often associated with reduced oral pain intensity, oral dysfunction, and a reduced need for pain medication (Table 4). In particular, studies [17,24] reporting the use of analgesic drugs showed a lower percentage of patients requiring non-opioid or opioid analgesics in the groups treated with ZnC compared to the control group. However, two RCTs [20,21] using a ZnC mouthwash and lozenge respectively reported no significant differences in oral pain outcomes compared to control group.

Oral function parameters, such as the need for nutritional support, dysgeusia, or xerostomia, were reported in four studies [20,21,23,24]. In pediatric patients undergoing HSCT [24], the use of ZnC was associated with a shorter duration of parenteral nutritional support (11.1 days vs. 24.3 days, *p* = 0.016), suggesting a reduced functional impact of oral mucositis. No significant differences were observed in the adult population in one study [20], while the study by Tsubura-Okubo et al. [23] showed lower rates of oral pain (26.6% vs. 75.6%, *p* < 0.001) and dysgeusia (13.9% vs. 36.6%, *p* = 0.004). In addition, Kitagawa et al. [21] reported scores of xerostomia (27.7% vs. 31.7%, *p* = 0.678) and dysgeusia (59.6% vs. 51.2%, *p* = 0.431) not significantly different between prevention and control group.

In terms of safety, ZnC has been well tolerated. No serious adverse events attributable to ZnC were reported in any of the studies reviewed.

### 3.10. Grading of Recommendations, Assessment, Development, and Evaluation (GRADE)—Certainty of Evidence

A formal GRADE assessment was performed for all three primary outcome domains (Table 5). The overall certainty of evidence was rated as low for severe oral mucositis (grade ≥ 3) and oral mucositis grade ≥ 2 and very low for oral mucosal healing. The principal reasons for downgrading were: serious risk of bias in the majority of included studies; substantial inconsistency (particularly for grade ≥ 2 outcomes, I^2^ = 80.1%); clinical and methodological indirectness arising from heterogeneity of populations, settings, and formulations; and imprecision of the pooled estimates under random-effects models, with confidence intervals crossing the null for both mucositis outcomes. For oral mucosal healing, direct evidence was limited to a single small prospective study (*n* = 10), with remaining data derived from indirect recovery indicators, resulting in very low certainty. These ratings are consistent with the cautious interpretation adopted throughout this review and further underscore the need for well-designed, adequately powered, randomized controlled trials with pre-specified, homogeneous outcomes.

## 4. Discussion

This systematic review evaluated the efficacy of zinc L-carnosine (ZnC) complex both in promoting mucosal healing and as a protective agent in various clinical settings, including patients undergoing hematopoietic stem cell transplantation (HSCT), head and neck chemoradiotherapy/radiotherapy (CRT/RT), and oral surgery. Overall, our findings suggests that ZnC may be associated with beneficial effects in reducing the incidence/severity of oral mucositis, controlling pain, and improving surgical wound healing. However, the data observed are characterized by high methodological heterogeneity, a limited number of randomized clinical trials, and the presence of bias in the studies analyzed, which reduce the strength of the evidence described.

The results of the meta-analysis confirm the results of the qualitative analysis of the individual studies. Regarding the incidence of severe mucositis (≥3), the odds ratio calculated using the Common Effect model highlighted significant value (OR 0.48; 95% CI 0.32–0.72). However, statistical significance was not maintained under the random-effects model, indicating that the observed effect should be interpreted with caution. The same result was observed about the onset of moderate mucositis (≥2), where the preventive effect of ZnC were significant (OR 0.56; 95% CI 0.33–0.95). In the random-effects model, significance was not maintained. This data may be explained by the clinical and methodological differences among the included studies. In particular, the study by Nakagaki et al. [20] differed from most of the included studies in that ZnC was administered as a simple mouthwash rather than as a mucoadhesive formulation, such as suspensions or lozenges, which allow for more prolonged contact with the mucosa.

The GRADE assessment further supports a cautious interpretation of the findings, with low-certainty evidence for oral mucositis outcomes (grade ≥ 2 and grade ≥ 3) and very low-certainty evidence for oral mucosal healing, mainly due to methodological limitations, study heterogeneity, and imprecision of the available estimates.

The clinical context most described concerns patients undergoing HSCT. Various observational studies and a randomized multicenter clinical trial show a positive effect in reducing the incidence of oral mucositis, specifically when used for therapeutic purposes, i.e., in conjunction with conditioning chemotherapy prior to stem cell transplantation. Several studies reporting favorable outcomes used formulations that promote prolonged contact with the oral mucosa, such as mucoadhesive suspensions based on sodium alginate, polyacrylic acid, carboxyvinyl polymer, which increase the local effect of ZnC. Kitagawa et al. [21], in their randomized multicenter study, showed a statistically significant reduction in preventing the onset of oral mucositis (≥2), but not in severe mucositis (≥3), suggesting a positive effect especially in limiting the onset of mucosal damage rather than reducing the progression of more severe stages. Furthermore, the assessment of outcomes related to pain perception, the consequent use of opioid and non-opioid drugs, and the impact on oral function (e.g., xerostomia and the need for parenteral nutrition) showed encouraging results in the group of patients treated with ZnC, suggesting a significant clinical impact in the management of patients with oral mucositis. In fact, the loss of integrity of the oral barrier allows bacteria to enter the bloodstream and promotes the activation of exogenous danger signals, amplifying systemic inflammation with potential worsening of acute GVHD [23]. Furthermore, Greenberg et al. [26] reported a significantly lower incidence of sepsis related to oral infections in patients who underwent professional oral management prior to chemotherapy compared to those who did not (25% versus 77%), emphasizing the clinical importance of mucosal integrity. In contrast, the randomized ToPaz study [20] showed no significant difference between ZnC and sodium bicarbonate in preventing the onset of oral mucositis in HSCT patients. However, these differences could be associated with the use of different formulations, modes of administration, outcome assessment, and the use of an active comparator that could have attenuated the differences between the two treatments. In fact, the ToPaz study differs in that it administered a ZnC-based mouthwash rather than a mucoadhesive formulation or ingested lozenges.

In patients with head and neck cancer treated with CRT/RT, the studies analyzed suggest a possible beneficial effect of the ZnC compound in reducing the onset of mucosal damage, accelerating mucosal recovery, and indirectly reducing treatment and hospitalization times—in particular with the use of mucoadhesive formulations that increase the contact of the active ingredient with the damaged mucosa. These results suggest that the prevention of mucositis may have not only clinical but also economic consequences. However, the data analyzed derive from non-randomized studies or retrospective analyses, which present an intrinsic risk of bias and do not allow definitive conclusions to be drawn in this specific setting.

With regard to oral surgery, a single prospective study conducted on 10 patients analyzed the effect of ZnC in improving the quality of mucosal healing. The study data showed higher PHI and MHI scores for the wound at 21 and 28 days, with no significant differences observed in the initial stages [22]. These data suggest a possible beneficial effect in the proliferation and tissue remodeling phases, rather than in the early healing processes.

The clinical results described in the studies analyzed in this systematic review are consistent with the known biological mechanisms of ZnC [27]. In fact, its anti-inflammatory, antioxidant, and cytoprotective properties have been associated with increased cell migration and proliferation, which are essential steps in the mucosal healing process [15]. Zinc is considered to have a direct role as an antioxidant and as a modulator of the expression of other antioxidant systems [28]. L-carnosine, in addition to enhancing the absorption and release of zinc in target tissues, acts as a free radical scavenger, buffer, and superoxide dismutase (SOD)-like agent, contributing to the protection of damaged tissue [15]. Numerous preclinical studies have shown that ZnC exerts a dose-dependent anti-inflammatory and antioxidant effect. In particular, it reduces the expression of pro-inflammatory cytokines (TNF-α, IL-1β, IL-6, IL-8), inhibits the activation of NF-κB, and decreases iNOS, while increasing the expression of growth factors (VEGF, bFGF, PDGF) and antioxidant enzymes such as SOD and HO-1 [29,30]. In addition, it stimulates the expression of heat shock proteins (HSP), contributing to cell protection and modulation of the inflammatory response [31]. Furthermore, the gene expression of Insulin-like Growth Factor (IGF)-1, a polypeptide significantly involved in cell proliferation and differentiation processes, was found to be increased following stimulation with ZnC in endothelial cells or fibroblasts cultured in vitro [32]. From a pathophysiological point of view, chemoradiotherapy-induced oral mucositis is characterized by an initial phase of oxidative damage mediated by reactive oxygen species, followed by NF-κB activation, release of pro-inflammatory cytokines (TNF-α, IL-1β, IL-6), ulceration, and a subsequent healing phase [33]. Several treatments have been proposed, but unfortunately management remains a difficult challenge [7]. The combined antioxidant, anti-inflammatory, and proliferation action of ZnC appears consistent with the pathogenetic mechanisms of mucositis, providing a biological rationale for its use as a preventive or therapeutic treatment [34,35]. In addition to oral mucositis, ZnC is widely studied in gastrointestinal diseases, where it acts locally on damaged mucosa [16]. Preclinical and clinical studies have demonstrated improved healing of peptic ulcers [16], a possible increase in Helicobacter pylori eradication rates when combined with triple therapy [36], a protective effect in NSAID-induced enteropathy [37], lesions induced by endoscopic submucosal dissection [38], colitis [39], and hemorrhoidal disease [40].

The results of this systematic review should be interpreted with caution. Only two studies were randomized clinical trials, while the remaining studies were prospective or retrospective cohorts characterized by moderate or severe risk of bias. The critical point, however, concerns the substantial clinical and methodological heterogeneity among the included studies in terms of population (adults or children), clinical settings, ZnC formulation, method of administration, type of therapy (preventive or therapeutic treatment), and outcomes analyzed. This makes it more difficult to perform a comparative analysis of the studies and an accurate summary of the clinical benefits. In addition, publication bias cannot be excluded. Although exploratory funnel plot inspection and Egger’s regression tests did not suggest substantial asymmetry, the small number of included studies limits the reliability of these assessments. Furthermore, the available literature is predominantly composed of studies reporting positive or trend-positive findings, and neutral or negative results may be underrepresented.

Despite the limitations mentioned above, this review highlights some important clinical considerations. First, in all the studies analyzed, the ZnC compound was well tolerated and did not cause any serious adverse events in the patients to whom it was administered. The most clinically relevant finding, confirmed by meta-analysis, concerns the reduction in the incidence of oral mucositis, together with a reduction in painful symptoms, the need for opioid and non-opioid medications, and a positive impact on oral function, assessed by the need for parenteral nutrition in addition to clinical parameters such as dysgeusia or xerostomia. Another important clinical consideration concerns the need for physicians to implement the best measures to safeguard mucosal integrity, especially in patients requiring HSCT, as a loss of mucosal continuity is associated with an increased possibility of microbes passing into the bloodstream, contributing to amplified systemic inflammation with significant negative repercussions for patients. In this regard, the ZnC compound may contribute to promoting the maintenance of mucosal integrity, suggesting its clinical use as a complement to standard oral care protocols.

One relevant aspect may be the formulation of ZnC. In fact, better clinical results were observed in those who used mucoadhesive suspensions or lozenges, which allowed prolonged contact of the active ingredient with the mucosa. However, direct comparisons between formulations are lacking. Therefore, any apparent differences between formulations should be interpreted with caution.

To define the actual protective and healing effect of ZnC in different clinical settings, further randomized clinical trials are needed, with well-defined and homogeneous outcomes. In particular, it would be appropriate to define clear protocols both in preventive and in therapeutic contexts, comparing different types of formulations and including endpoints that assess quality of life, pain assessment scales (e.g., VAS/NRS scale), volumetric reductions in the damaged mucosal area, and the need to take analgesic drugs. Further research focused on the biological mechanisms of the regenerative and cytoprotective effect of ZnC on oral mucosal cells could help to better define the role of this compound in the management of high-risk patients.

## 5. Conclusions

In conclusion, the results of this systematic review suggest that zinc L-carnosine is a compound that is well tolerated by patients and has clinical potential for healing and protecting the oral mucosa. The results of meta-analysis suggest a favorable effect in reducing the incidence of oral mucositis, pain control, and improved quality of surgical wound healing, with no serious side effects. However, these findings should be interpreted cautiously, as statistical significance was not maintained under random-effects models and substantial heterogeneity was observed across studies. Thus, further randomized controlled clinical trials with high quality protocols are needed to confirm these preliminary results and better define the clinical potential of zinc L-carnosine in the management of the oral mucosa damage.

## Figures and Tables

**Table 1:** Characteristics of the included studies (*n* = 544 subjects. RCT: randomized controlled trial; NA: not applicable; HSCT: hematopoietic stem cell transplantation; RT/CRT: radiotherapy or chemoradiotherapy: OM: Oral Mucositis.

Author (Year)	Study Design	Population (*n*)	Age (Years)	Clinical Setting	ZnC Formulation	Control	Primary Outcome
Nakagaki et al. 2024 [20]	RCT	108 adults	NA	HSCT	Mouthwash	Sodium bicarbonate	OM prevention
Dell’Olio et al. 2023 [22]	Prospective cohort	10 adults	44.6 ± 19.2	Oral surgery	Sodium alginate-based suspension	Standard oral care	Promoting oral surgical wound healing
Kitagawa et al. 2021 [21]	RCT	88 adults	18–90	HSCT	Lozenge	Delayed ZnC	OM prevention
Tsubura-Okubo et al. 2021 [23]	Retrospective cohort	120 adults	NR	HSCT	Adhesive ZnC solution (PPAA)	Standard oral care	OM prevention
Funato et al. 2018 [24]	Retrospective cohort	16 children	1–18	Pediatric HSCT	Sodium alginate-based suspension	Azulene gargle	OM prevention
Hayashi et al. 2016 [17]	Prospective cohort	66 adults	NA	HSCT	Suspension/Lozenge	Standard oral care	OM prevention
Suzuki et al. 2016 [6]	Retrospective cohort	104 adults	NA	RT	Sodium alginate-based suspension	Azulene gargle	Reduction of OM severity during RT
Doi et al. 2015 [25]	Prospective + retrospective	32 adults	NA	RT/CRT	Mucoadhesive ZnC (carboxyvinyl polymer-based)	Standard oral care	OM prevention

**Table 2:** Effects of zinc L-carnosine on oral mucosal protection. OM: oral mucositis; ZnC: Zinc L-carnosine; HSCT: hematopoietic stem cell transplantation; RT/CRT: radiotherapy/chemoradiotherapy; NA: not applicable; CTCAE: Common Terminology Criteria for Adverse Events; WHO: World Health Organization.

Author (Year)	Clinical Setting	ZnC Formulation	Control	OM Grading Scale	Incidence of OM	Incidence of Severe OM (Grade ≥ 3)
Nakagaki et al. 2024 [20]	HSCT	Mouthwash	Sodium bicarbonate	WHO	Grade 2–4: 72% vs. 62% (NS)	Grade 3–4: 35% vs. 36% (NS)
Kitagawa et al. 2021 [21]	HSCT	Lozenge	Delayed ZnC	CTCAE	Grade ≥ 2: 22.0% vs. 44.7%, *p* = 0.025	Grade ≥ 3: 14.6% vs. 10.6% (NS)
Tsubura-Okubo et al. 2021 [23]	HSCT	Adhesive ZnC solution (PPAA)	Standard oral care	CTCAE	NA	Grade ≥ 3: 15.2% vs. 36.6%, *p* = 0.008
Funato et al. 2018 [24]	Pediatric HSCT	Sodium alginate-based suspension	Azulene gargle	WHO	NA	Grade ≥ 3: 20% vs. 83.3%, *p* = 0.035
Hayashi et al. 2016 [17]	HSCT	Suspension/Lozenge	Oral standard care	CTCAE	Grade ≥ 2: 22.6% (suspension)/12.5% (lozenge) vs. 73.7% (control), *p* < 0.01	Grade 3: 3.2–6.3% vs. 21.1% (NS)
Suzuki et al. 2016 [6]	RT	Sodium alginate-based suspension	Azulene gargle	CTCAE	69.6% vs. 88.0% (NS)	Grade ≥ 3: 16.5% vs. 52%, *p* = 0.0003
Doi et al. 2015 [25]	RT/CRT	Mucoadhesive ZnC (carboxyvinyl polymer-based)	Standard oral care	CTCAE	NA	Grade ≥ 3: 29% vs. 40% (NS)

**Table 3:** Effects of zinc L-carnosine on oral mucosal healing. NA: not applicable; HSCT: hematopoietic stem cell transplantation; RT/CRT: radiotherapy or chemoradiotherapy; OM: Oral Mucositis.

Author (Year)	Clinical Setting	ZnC Formulation	Control	Healing/Recovery Outcome	Main Outcomes
Dell’Olio et al. 2023 [22]	Oral surgery	Sodium alginate-based suspension	Standard oral care	Modified Landry’s Healing Index (MHI) and Percentage Healing Index (PHI)	Higher MHI e PHI scores at 21 and 28 days (MHI: *p* = 0.026, *p* = 0.048; PHI: *p* = 0.029, *p* = 0.007)
Kitagawa et al. 2021 [21]	HSCT	Lozenge	Delayed ZnC	Progression to severe OM (≥3)	Lower progression to severe OM; low incidence rate of OM ≥ 3 (10.6%)
Tsubura-Okubo et al. 2021 [23]	HSCT	Adhesive ZnC solution (PPAA)	Standard oral care	Not directly assessed	Lower oral pain and dysgeusia (*p* < 0.001, *p* = 0.004)
Doi et al. 2015 [25]	RT/CRT	Mucoadhesive ZnC (carboxyvinyl polymer-based)	Standard oral care	Time to clinical resolution of OM	Faster resolution of OM in the ZnC group (~3 vs. ~5–6 weeks—graphical estimate)

**Table 4:** Secondary outcomes related to pain, oral function, and adverse events. NA: not applicable; HSCT: hematopoietic stem cell transplantation; RT/CRT: radiotherapy or chemoradiotherapy; SD: statistically significant difference.

Author (Year)	Clinical Setting	ZnC Formulation	Pain Assessment and Analgesic Use	Oral Function	Adverse Events
Nakagaki et al. 2024 [20]	HSCT	Mouthwash	No SD in oral pain	No SD in oral function	No adverse events reported
Kitagawa et al. 2021 [21]	HSCT	Lozenge	No SD in analgesic use	No SD regarding Xerostomia (27.7% vs. 31.7%, *p* = 0.678) and dysgeusia (59.6% vs. 51.2%, *p* = 0.431)	No adverse events reported
Tsubura-Okubo et al. 2021 [23]	HSCT	Adhesive ZnC solution (PPAA)	Lower oral pain scores (26.6% vs. 75.6%, *p* < 0.001)	Lower dysgeusia scores (13.9% vs. 36.6%, *p* = 0.004)	No adverse events reported
Funato et al. 2018 [24]	Pediatric HSCT	Sodium alginate-based suspension	Reduced need for opioid analgesics (30% vs. 100%, *p* = 0.011)	Shorter duration of parenteral nutrition (11.1 days vs. 24.3 days, *p* = 0.016)	No adverse events reported
Hayashi et al. 2016 [17]	HSCT	Suspension/Lozenge	Reduced need for non-opioid analgesics (16.1% suspension; 12.5% lozenge vs. 89.5%, *p* < 0.01)	NA	No adverse events reported
Suzuki et al. 2016 [6]	RT	Sodium alginate-based suspension	NA	NA	No adverse events reported
Doi et al. 2015 [25]	RT/CRT	Mucoadhesive ZnC (carboxyvinyl polymer-based)	NA	NA	No adverse events reported

**Table 5:** GRADE Summary of Findings.

Outcome	Evidence Base	Effect	Risk of Bias	Inconsistency/Indirectness	Imprecision	Publication Bias	Certainty	Suggested Conclusion
Severe oral mucositis (grade ≥ 3)	7 studies, 532 participants; RCTs plus non-randomized studies	Common-effect OR 0.48 (95% CI 0.32–0.72); random-effects OR 0.44 (95% CI 0.18–1.06); I^2^ = 56.1%	Serious: most evidence from non-randomized studies; RCTs with some concerns.	Serious: heterogeneous populations, clinical settings and ZnC formulations.	Serious: random-effects CI crosses no effect.	Undetected but strongly suspected/uncertain: k = 7, funnel/Egger underpowered.	Low	Favorable signal, but not robust under random-effects model and limited by bias/heterogeneity.
Oral mucositis (grade ≥ 2)	4 studies, 335 participants	Common-effect OR 0.56 (95% CI 0.33–0.95); random-effects OR 0.36 (95% CI 0.10–1.29); I^2^ = 80.1%	Serious: mixture of study designs and risk-of-bias concerns.	Very serious: high heterogeneity and formulation differences.	Serious: random-effects CI wide and crosses no effect.	Undetected but not assessable: k = 4.	Low	Suggestive reduction, but certainty is substantially limited.
Oral mucosal healing/wound healing	Mainly one small prospective oral-surgery study plus indirect recovery outcomes	Higher MHI/PHI at 21 and 28 days in one small study; other outcomes indirect and heterogeneous.	Very serious: very small sample and non-randomized evidence.	Serious: only one direct oral-surgery study; indirect evidence from OM recovery.	Serious: small numbers and imprecise estimates.	Not assessable.	Very low	Hypothesis-generating only.

## Data Availability

The original contributions presented in this study are included in the article and Supplementary Material. Further inquiries can be directed to the corresponding author.

## Supplementary Materials

The following supporting information can be downloaded at: https://www.mdpi.com/article/10.3390/dj14070408/s1, PRISMA 2020 checklist [41].
